# Evidence for host–microbiome co‐evolution in apple

**DOI:** 10.1111/nph.17820

**Published:** 2021-11-25

**Authors:** Ahmed Abdelfattah, Ayco J. M. Tack, Birgit Wasserman, Jia Liu, Gabriele Berg, John Norelli, Samir Droby, Michael Wisniewski

**Affiliations:** ^1^ Institute of Environmental Biotechnology Graz University of Technology Petersgasse 12 Graz 8010 Austria; ^2^ Leibniz Institute for Agricultural Engineering and Bioeconomy (ATB) Max‐Eyth Allee 100 14469 Potsdam Germany; ^3^ Department of Ecology, Environment and Plant Sciences Stockholm University Svante Arrhenius väg 20A Stockholm SE‐106 91 Sweden; ^4^ Chongqing Key Laboratory of Economic Plant Biotechnology College of Landscape Architecture and Life Sciences Chongqing University of Arts and Sciences Yongchuan Chongquing 402160 China; ^5^ Institute for Biochemistry and Biology University of Postdam 14476 Potsdam OT Golm Germany; ^6^ Appalachian Fruit Research Station United States Department of Agriculture – Agricultural Research Service Kearneysville WV 25430 USA; ^7^ Department of Postharvest Science Agricultural Research Organization The Volcani Institute PO Box 15159 Rishon LeZion 7505101 Israel; ^8^ Department of Biological Sciences Virginia Polytechnic Institute and State University 220 Ag Quad Ln Blacksburg VA 24061 USA

**Keywords:** bacterial community, endophytes, fungal community, microbial introgression, microbiota, phylosymbiosis

## Abstract

- Plants evolved in association with a diverse community of microorganisms. The effect of plant phylogeny and domestication on host–microbiome co‐evolutionary dynamics are poorly understood.
- Here we examined the effect of domestication and plant lineage on the composition of the endophytic microbiome of 11 *Malus* species, representing three major groups: domesticated apple (*M*. *domestica*), wild apple progenitors, and wild *Malus* species.
- The endophytic community of *M*. *domestica* and its wild progenitors showed higher microbial diversity and abundance than wild *Malus* species. Heirloom and modern cultivars harbored a distinct community composition, though the difference was not significant. A community‐wide Bayesian model revealed that the endophytic microbiome of domesticated apple is an admixture of its wild progenitors, with clear evidence for microbiome introgression, especially for the bacterial community. We observed a significant correlation between the evolutionary distance of *Malus* species and their microbiome.
- This study supports co‐evolution between *Malus* species and their microbiome during domestication. This finding has major implications for future breeding programs and our understanding of the evolution of plants and their microbiomes.

## Introduction

The evolution of plants has occurred with diverse microbial communities inhabiting their tissues (Yeoh *et al*., 2017; Delaux & Schornack, 2021). Collectively called the plant microbiome, these microorganisms fulfill important functions for their host’s health by manipulating its gene expression, hormonal pathways and increasing its tolerance to biotic and/or abiotic stresses (Berg *et al*., 2020). Empirical evidence shows that the phenotypic expression of host traits is due to the combined genetic expression of the host and the host‐associated microbiome (Matsumoto *et al*., 2021; Ravanbakhsh *et al*., 2021).

The long and intimate history of plant–microbiome associations, the importance of host genetics in shaping the microbiome, and the profound effect that microbiomes have on the traits of their hosts indicate that plants and their associated microbiomes are co‐evolving (Krings *et al*., 2007; Delaux & Schornack, 2021). Such co‐evolutionary dynamics may be reflected in a tendency of closely related plant species to host similar microbial communities, also known as phylosymbiosis (Brucker & Bordenstein, 2013; Theis *et al*., 2016; Mazel *et al*., 2018). Phylosymbiosis has been demonstrated in several plant groups, with stronger phylosymbiotic patterns for endophytic than epiphytic or rhizosphere‐associated microbiomes (Bouffaud *et al*., 2014; Schlaeppi *et al*., 2014; Vincent *et al*., 2016; Mazel *et al*., 2018; Mendes *et al*., 2018; Abdullaeva *et al*., 2020; Kim *et al*., 2020). These observations were suggested to be governed by two mutually nonexclusive mechanisms, namely heritability and inheritance (Peiffer *et al*., 2013; Beilsmith *et al*., 2019). Heritability refers to how the host genotype affects the assembly of the plant microbiome from the environment (Beilsmith *et al*., 2019; Wagner, 2021), and inheritance refers to the microbial community that is vertically transmitted to subsequenct generations via seeds (Beilsmith *et al*., 2019; Abdelfattah *et al*., 2021b; Wagner, 2021). In this context, it is important to understand the impact of domestication on the co‐evolutionary dynamics between plants and their microbiomes, and test whether phylosymbiotic patterns extend from the phylogeny of wild species to their domesticated progenies.

Domestication and breeding history can have a major impact on the diversity, abundance, and composition of the plant microbiome. Some domesticated plants were found to have a distinct microbial community composition and a lower capacity to interact with microbial symbionts, as compared to their wild relatives (Mutch & Young, 2004; Kiers *et al*., 2007; Leff *et al*., 2017; Pérez‐Jaramillo *et al*., 2018; Porter & Sachs, 2020; Favela *et al*., 2021). The majority of studies have shown a decrease in microbial species diversity with domestication (Bulgarelli *et al*., 2015; Coleman‐Derr *et al*., 2016). However, others have reported increased diversity, e.g. in lettuce, cereal seeds (wheat and barley) and common bean (Cardinale *et al*., 2015; Abdullaeva *et al*., 2020), or no effect as in the case of wheat and sunflower (Leff *et al*., 2017; Spor *et al*., 2020). Among domesticated plants, phylosymbiosis has been observed, for example in Poaceae roots, seeds, and rhizosphere (Bouffaud *et al*., 2014; Abdullaeva *et al*., 2020; Favela *et al*., 2021). Moreover, recent studies on microbiomes of breeding lines showed that hybrid plants share a large fraction of their bacterial community with their parents as for example in the case of Cucurbita seeds and apple shoot endophytes (Adam *et al*., 2018; Liu *et al*., 2018; Kusstatscher *et al*., 2021a). Yet, it is unclear if the propotional contrubution of the microbiome from parents to offpring correspond to amount of genetic material contributed by each parent during breeding and/or domestication.

One particularly suitable system to explore the impact of domestication on the diversity and composition of the microbiome, as well as phylosymbiosis, is apple. Apple (*Malus × domestica*), was primarily domesticated about 4000–10 000 yr ago from *Malus sieversii* (Ldb.) Roem, whose center of origin is the Tian Shan Mountains of Central Asia. Apple germplasm thereafter moved westwards by people traveling along the Silk Route (Cornille *et al*., 2012; Duan *et al*., 2017), during which time other *Malus* species, such as *M. prunifolia* (Willd.) Borkh in Asia, *M. orientalis* Uglitz. in the Caucasus, and *M*. *sylvestris* Mill. in Europe contributed to the genome of *M. domestica* through introgressive hybridization (Cornille *et al*., 2014; Volk *et al*., 2015; Volk, 2019). The domestication of apple has resulted in thousands of cultivars with large fruits, high yield, firm flesh, and high sugar content (Duan *et al*., 2017). Heirloom cultivars are older varieties for which genetic pedigree information is lacking, are known to have existed for long periods of time but have not necessarily served as founding genotypes for the more modern cultivars that have been developed. The microbiome of domesticated apple is highly diverse, and has been shown to be affected by plant genotype, management practices, soil composition, postharvest treatments, geographical location, and health status (Shade *et al*., 2013; Abdelfattah *et al*., 2016, 2020, 2021a; Shen *et al*., 2018; Wassermann *et al*., 2019a, 2019b,2019a, 2019b; Cui *et al*., 2021; Whitehead *et al*., 2021). Little is known, however, about the composition of the endophytic microbiome of wild *Malus* species or the impact of domestication on the microbiome of *M*. *domestica* and its many cultivars.

The current study examined the endophytic microbial communities of *Malus* species collected from the United States Department of Agriculture – Agricultural Research Service (USDA‐ARS) Apple Germplasm Repository in Geneva, NY, USA. The common garden setup was used to overcome geographical variation in environmental factors, such as soil properties, environmental microbiome, and climatic conditions. The study focused on endophytes, since they are less likely to be environmental contaminants than epiphytes and are expected to have the most intimate relationship with their host (Hardoim *et al*., 2015). The microbial communities were identified using amplicon sequencing and their abundance was quantified by quantitative real‐time polymerase chain reaction (qPCR). The aims of the study were to:

1. investigate the effect of apple domestication on the diversity, abundance and composition of the endophytic microbial community by comparing domesticated apple to wild progenitor and wild *Malus* species, and by comparing heirloom and modern cultivars
2. determine whether phylosymbiotic patterns can be observed among *Malus* species
3. determine the origin of the *M. domestica* microbiome by estimating the proportional contribution of wild *Malus* species to domesticated apple

**Fig. 1:**
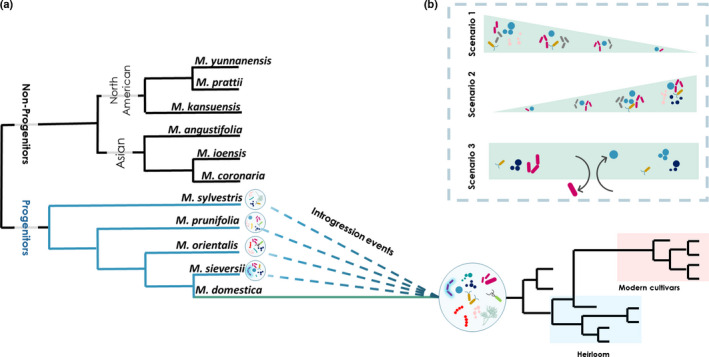
A conceptual figure on the impact of domestication on the plant endophytic microbiome. (a) A phylogenetic distance among *Malus* species which contains wild species (black branches) and progenitor wild species (blue branches). The extended green branch represents *Malus domestica* with its close affiliation its main ancestor (*M. sieversii*). Dashed lines indicate introgression events between *Malus* progenitors which contributed to the formation of *M. domestica*. (b) The predicted three scenarios: Scenario 1, reduction in species diversity due to loss in microbial species; Scenario 2, increase in microbial diversity due to introgressive hybridization during the apple domestication; Scenario 3, diversity was not affected by domestication.

Regarding species diversity, richness, and evenness, we assumed three scenarios to be equally likely during apple domestication (Scenarios 1–3 in Fig. 1). A reduction in species interactions has been associated with domestication, which would result in a less diverse microbiome in *M*. *domestica* than in wild *Malus* species (Mutch & Young, 2004; Kiers *et al*., 2007; Leff *et al*., 2017; Pérez‐Jaramillo *et al*., 2018; Porter & Sachs, 2020; Favela *et al*., 2021) (Scenario 1 in Fig. 1). Secondly, introgressive hybridization during the domestication of apple may have been accompanied by the introgression of the microbiome, resulting in higher diversity in *M. domestica* (Cardinale *et al*., 2015; Abdullaeva *et al*., 2020) (Scenario 2 in Fig. 1). Lastly, the microbiome may have been reshuffled during domestication, without an increase or decrease in the number of species (Scenario 3 in Fig. 1). Importantly, these scenarios are not mutually exclusive, and processes within each scenario could counteract or complement each other. Similar scenarios are expected to play out in the case of heirloom and modern cultivars. We also expected to find a strong correlation between the phylogenetic distance among *Malus* species and the community composition of their associated microbiomes (Bouffaud *et al*., 2014; Schlaeppi *et al*., 2014; Vincent *et al*., 2016) (Fig. 1). Specifically, we anticipated (1) the *M. domestica* microbiome to be more similar to its wild progenitors, especially to its main ancestor *M. sieversii*, than to wild, nonprogenitor species; (2) a correlation between the genetic distance of wild species of North American and Asian origin and their microbial community composition; and (3) that the introgression events consisted of both genetic and microbial contributions, which together shaped the microbiome of the domesticated apple (Fig. 1).

## Materials and Methods

### Plant material and experimental design

Samples were collected from an orchard of the Apple Germplasm Repository of the USDA‐ARS, located in Geneva, NY, USA, to limit the effects of geographical location or climate on the microbiome. The orchard is characterized by high lime developed soil on glacial till with an average annual temperature of 9.4°C and rainfall of 89 cm. The selection of accessions was based on chloroplast haplotypes classification reported by Volk *et al*. (2015), in which the accessions were grouped into three clades: North American, Asian, and *M. domestica* admixture (Supporting Information Table S1). The present experiment was set up to include 61 apple accessions from 11 *Malus* species, representing three major groups: (1) domesticated apple cultivars (*M*. *domestica*), (2) wild progenitors of domesticated apple, and (3) nonprogenitors wild *Malus* species that did not contribute to apple domestication. While both the wild progenitors and the nonprogenitors represent wild apple species, for the purpose of simplicity and readability, we will use the term ‘wild’ only for the nonprogenitor species. The domesticated apple was represented by 18 accessions, representing seven heirloom cultivars (Fenouillet Gris, Miron Sacharanij, Coat Jersey, Landsberger Reinette, Borowitsky, Taylors, Fillbarrel) and six modern cultivars (Delicious, Golden Delicious, Honeycrisp, Northern Spy, Splendour, Frostbite). Apple wild progenitors included *M. sieversii* (nine accessions), *M. sylvestris* (three accessions), *M. orientalis* (seven accessions) and *M. prunifolia* (six accessions). Lastly, the wild *Malus* species consisted of *M. kansuensis* (three accessions), *M. prattii* (three accessions), *M. yunnanensis* (three accessions), *M. angustifolia* (three accessions), *M. ioensis* (three accessions), and *M. coronaria* (three accessions). The majority of the collected accessions were originally planted as seeds, self‐rooted cuttings or grafted onto EMLA7 (Table S1).

### Collection and processing of samples

Shoots, *c*. 7–10 mm in diameter and 15 cm in length, were collected on November 2018 from current‐year growth. Four shoots of every accession were surface sterilized with 5% sodium hypochlorite (v/v) and rinsed three times in sterile water. The bark was removed with a sterile razor, as described in Liu *et al*. (2018). After peeling the bark, shoots were cut into 0.5–1.0 cm segments, and transferred to 50‐ml Falcon tubes with 40 ml sterile double distilled water (ddH_2_O) and shaken horizontally at 200 rpm for 30 min. The suspension was then transferred to centrifuge tubes and centrifuged at 12 000 ***g*** for 30 min. Pelleted solutions were used for DNA extraction with DNAeasy PowerSoil Kit (Qiagen, Hilden, Germany) according to the manufacturer’s instruction. DNA quality and integrity were assessed using a NanoDrop UV‐spectrophotometer (ThermoFisher Inc., Grand Island, NY, USA). The same DNA extracts were used for amplicon library preparation and qPCR measurements.

### Library preparation and sequencing

Library preparation and sequencing was conducted as previously described (Abdelfattah *et al*., 2020). Briefly, bacterial 16S rDNA region was amplified using the universal primers 515F and 806R in conjunction with peptide nucleic acids (PNAs) added to reduce the amplification of plant chloroplast and mitochondrial sequences. For fungal amplification, internal transcribed spacer (ITS) amplicons were produced using ITS3/KYO2 and ITS4 primers along with a custom‐designed blocking oligo designed to inhibit amplification of host apple sequences. For prokaryote 16S and fungal ITS amplicon generation, PCR reactions were conducted in a total volume of 25 μl containing 12.5 μl of KAPA HiFi HotStart ReadyMix (Kapa Biosystems, Wilmington, MA, USA), 1.0 μl of each primer (10 μM), and 2.5 μl of DNA template. For the 16S amplification, 2.5 μl of mitochondrial PNA (5 μM), 2.5 μl of plastid PNA (5 μM), and 3 μl nuclease‐free water, and in ITS amplification 1.0 μl of blocking oligo (10 μM) and 7 μl nuclease‐free water were added.

Reactions were incubated in a T100 thermal cycler (Bio‐Rad, Hercules, CA, USA) at 95°C for 5 min followed by 30 cycles of 95°C for 30 s, 78°C for 5 s, 55°C for 30 s, 72°C for 30 s, and a final extension step at 72°C for 5 min. For fungal (ITS) amplicon generation, at 95°C for 5 min followed by 30 cycles of 95°C for 30 s, 55°C for 30 s, 72°C for 30 s and concluding with a final extension at 72°C for 5 min. Library preparation following amplicon PCR was performed as specified in the Illumina 16S Metagenomic Sequencing Library Preparation guide as outlined in conjunction with the use of a Nextera Index Kit (Illumina, San Diego, CA, USA) containing 96 indexes. Subsequent library size, quality, and confirmation of the absence of adapter dimers was checked on an Agilent 2100 Bioanalyzer (Agilent Technologies, Santa Clara, CA, USA). Paired‐end sequencing of amplicons was done on an Illumina MiSeq (Illumina) sequencer with a V3 600‐cycle Reagent Kit (Illumina).

### Bioinformatic and statistical analyses

Demultiplexing, quality trimming of low‐quality reads and creation of amplicon sequence variants (ASV) were done using the default parameters in Dada2 algorithm as integrated in Callahan *et al*. (2016) and Bolyen *et al*. (2019). Taxonomic assignment of the ASVs was done using Blast algorithm against the Silva 138 and UNITE databases for 16S and ITS reads, respectively (Abarenkov *et al*., 2010; Quast *et al*., 2012). MetagenomeSeq’s cumulative sum scaling (CSS) (Paulson *et al*., 2013) was used to account for uneven sequencing depth and then used for downstream analyses including Bray–Curtis dissimilarity metrics (Bray & Curtis, 1957), hierarchical clustering analysis and permutational multivariate analysis of variance (PERMANOVA). The *rarefy_even_depth* function implemented in the R package phyloseq v.1.32.0 was used to resample the ITS and 16S ASV tables to an even library size of 5000 and 1000, respectively. The rarefied tables were then used to calculate fungal and bacterial diversity using Shannon index (McMurdie & Holmes, 2013).

To evaluate the effect of domestication on the richness, evenness, diversity, and composition of the fungal and bacterial community, we modeled each response variable as a function of the fixed effect ‘domestication group’ (domesticated, wild progenitor and wild species) and species identity. Models with the univariate response variables were implemented using the function *aov* in R Package stats v.4.0.1 in R v.3.6.2 (R Core Team, 2020). Group means were compared using Tukey multiple comparisons of means.

Community composition was modeled using the function *adonis2* (PERMANOVA) in the package vegan (Oksanen *et al*., 2007). Pairwise comparisons were made using Pairwise Multilevel Comparison R package pairwiseadonis (Arbizu, 2019). To understand whether the change in microbial community composition is due to species turnover or species loss or gain, we calculated community dissimilarity using binary Jaccard dissimilarity index for each domestication group and then partitioned the calculated indexes into species turnover (βJTU) and species gain or loss (βJNE) (Baselga & Orme, 2012). To understand the effect of domestication on the plant core microbiome, we first calculated the core microbiome after transforming the ASV table into compositional data, keeping ASVs present in at least 70% of all samples within each domestication category, i.e. *M. domestica*, wild progenitors, and wild species, using microbiome R package v.1.10.0 (Leo Lahti & Shetty, 2017). We then compare the number of fungal and bacterial ASVs among the three domestication groups to determine if they all share the same core species, increase, or decrease along the chronosequence of *Malus* germplasm.

To quantify the relationship between *Malus* phylogeny and its microbiome (Brooks *et al*., 2016), we first used hierarchical clustering based on Bray–Curtis dissimilarity distances with ‘average’ as the clustering method. This was performed using *hclust* in R package stats v.4.0.1 and the results were visualized using *fviz_dend* function in the R package factoextra v.1.0.7 (Bray & Curtis, 1957; Kassambara & Mundt, 2020). Second, to calculate phylogenetic distances among *Malus* species, the ITS regions of the investigated species in addition to *Pyrus communis* as outgroup, were retrieved from National Center for Biotechnology Information (NCBI), aligned using the MUSCLE algorithm (list of accessions with alignment is available in Table S2), and the phylogenetic distance was inferred by using the neighbor‐joining tree estimation in R package phangorn (Saitou & Nei, 1987; Schliep, 2010). To quantify the congruence between *Malus* phylogeny and the microbiome, the topologies of the constructed dendrograms of the fungal and bacterial community were compared to the phylogenetic tree of *Malus* species, using Procrustes test in vegan (Peres‐Neto & Jackson, 2001).

To estimate the potential contribution of *Malus* species to the microbiome of domesticated apple, we used SourceTracker2, a Bayesian approach originally developed to estimate the environmental sources of the microbial community (Knights *et al*., 2011). In this analysis, each *Malus* species was assigned as a potential source and *M. domestica* was assigned as the only sink. Both sources and sink were rarefied to 1500 reads per sample.

### Quantitative real‐time polymerase chain reaction

Bacterial and fungal abundance was measured by qPCR using the primer pairs ITS1–ITS2 for fungi (White *et al*., 1990) and 515f–927r for bacteria (Köberl *et al*., 2011). Reaction mixtures contained 5 μl KAPA SYBR Green, 0.5 μl (10 μM each) of each primer, 1 μl template DNA, adjusted with PCR‐grade water to a final volume of 10 µl. A Rotor‐Gene 6000 real‐time rotary analyzer (Corbett Research, Sydney, NSW, Australia) was used to detect fluorescence intensity using the following cycling conditions: bacteria: 95°C for 3 min, 30 cycles of 95°C for 5 s, 54°C for 20 s, 72°C for 5 s, and a final melt curve of 72–96°C; fungi: 95°C for 3 min, 40 cycles of 95°C for 5 s, 58°C for 35 s, 72°C for 5 s with a final melt at 72°C for 10 min and a final melt curve of 72–96°C. For each sample replicate, three individual qPCR runs were conducted, and standard curves were constructed to determine the efficiency and linear range of the assay. Intermittently occurring gene copy numbers that were detected in negative control samples were subtracted from the respective run. Nonparametric Kruskal–Wallis test was applied to calculate statistical differences in bacterial and fungal abundance between groups and the *P*‐values were corrected using Bonferroni multiple test correction (Kruskal & Wallis, 1952).

## Results

### Sequencing results and the microbiome composition of *Malus* species

MiSeq sequencing yielded a total of 5813 667 ITS and 609 147 16S high‐quality reads after the removal of chimeras, plant chloroplast and mitochondrial sequences, and low‐quality reads. The clean reads were assigned to 2693 ITS fungal ASVs and 2688 bacterial ASVs. The number of sequences varied among samples, ranging from 81 147 to 1902 518 ITS reads and 10 578 to 187 894 16S reads. The fungal community was dominated by Ascomycota (71.2%) and Basidiomycota (27.0%), in which the genera *Aureobasidium* (45.4%), an unidentified genus of Pleosporaceae (13%), and *Filobasidium* (9.2%), prevailed. The bacterial community was dominated by Proteobacteria (82.9%), Actinobacteria (10.5%), and Bacteroidetes (2.8%). The predominant genera were *Sphingomonas* (23.5%), *Pseudomonas* (15.5%), and *Methylobacterium‐Methylorubrum* (15%) (Fig. S1).

### The effect of domestication on the microbial community

**Fig. 2:**
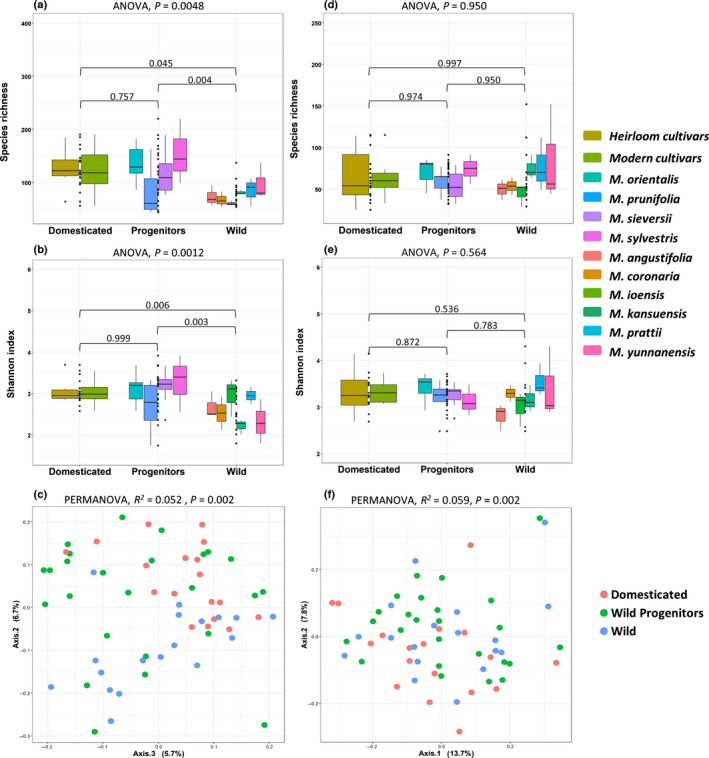
Box plots showing fungal (a–c) and bacterial (d–f) richness, Shannon diversity and community composition. The presented species were grouped into three groups from left to right: heirloom and modern cultivars of domesticated apple (*Malus* × *domestica*), wild progenitors (*M. orientalis*, *M. prunifolia*, *M. sieversii*, and *M. sylvestris*), and nonprogenitor *Malus* species (*M. angustifolia*, *M. coronaria*, *M. ioensis*, *M. kansuensis*, *M. prattii*, and *M. yunnanensis*). Superimposed on the box plots are the horizontally jittered raw data points combined for each domestication group. Box plots show the median (horizontal line), the lower and upper bounds of each box plot denote the first and third quartiles, and whiskers above and below the box plot show 1.5 times the interquartile range. The points located outside of the whiskers of the box plot represent the outliers. Ordination plots of fungal (c) and bacterial (f) community composition of *Malus* × *domestica*, wild progenitors and wild *Malus* species, based Bray–Curtis dissimilarity index. Results of the global statistical analyses are reported at the top of each panel and pairwise comparisons for alpha diversity are added onto the box plots.

Fungal species richness and diversity differed significantly among *M. domestica*, wild progenitors, and wild species (*P* = 0.0048 and *P* = 0.0012, respectively). Both richness and diversity were significantly higher in domesticated apple (*M. domestica*) and its wild progenitors than in wild species (Fig. 2a,b; Table S3). These estimates correspond also to absolute abundance of fungal gene copies numbers, measured via qPCR: endophytes of domesticated apple (*P* < 0.001) and its wild progenitors (*P* = 0.04) were significantly more abundant than those of wild apples, while no difference was found between progenitor and domesticated species (Fig. S2a; Table S4). The fungal community composition differed significantly among domesticated apple, progenitor species, and wild species (*R* ^2^ = 0.052, *P* = 0.001) (Fig. 2c), which was confirmed by pairwise comparisons (Table S4). Partitioning of beta diversity indicated that species turnover was the dominant factor in the differences observed in the fungal communities of wild, progenitor, and domesticated species (Fig. S2).

In contrast to fungi, bacterial species richness, evenness, and diversity did not differ among the domesticated apple, progenitor species, and wild species (Fig. 2d; Table S3). However, domestication had a significant effect on the bacterial community composition (*R* ^2^ = 0.059, *P* = 0.002) (Fig. 2e). Pairwise comparison showed that nonprogenitor wild species differed significantly from both wild progenitors and domesticated apples (*R* ^2^ = 0.047, *P* = 0.003, *R* ^2^ = 0.062, *P* = 0.003), whereas the comparison between wild progenitors and domesticated apple showed no significant differences (*R* ^2^ = 0.029, *P* = 0.123) (Table S4). Measurements of qPCR, however, revealed bacterial endophytes to be significantly less abundant in wild species compared to domesticated apples (*P* < 0.001); no difference in bacterial abundance was observed between the other domestication groups (Fig. S2b).

**Fig. 3:**
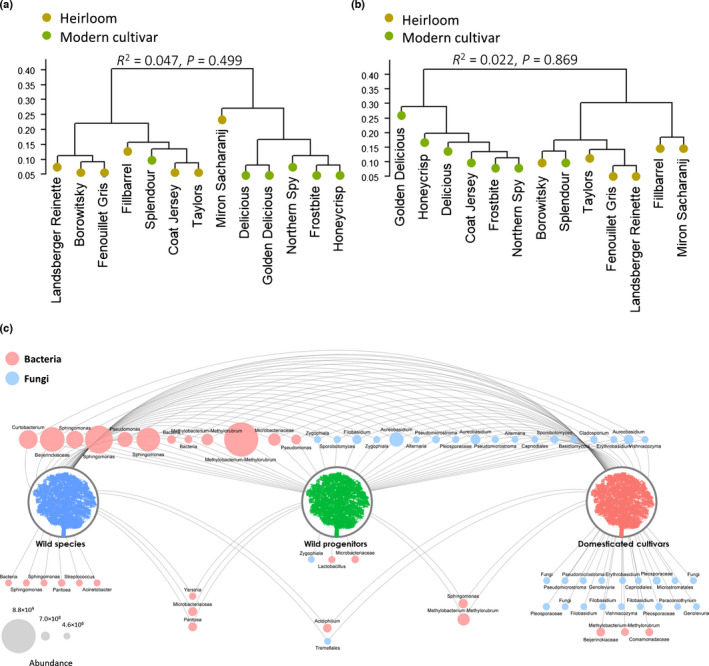
Dendrogram based on the similarity of the core fungal (a) and bacterial (b) community composition, according to Bray–Curtis index among *Malus domestica* cultivars, highlighting the difference between heirloom and modern cultivars. The dendrograms were visualized using the *fviz_dend* function in the R package factoextra v.1.0.7. Results of the global statistical analyses are reported at the top of each panel. (c) Network analysis showing the core microbiome distribution from wild *Malus* species, to progenitors, to domesticated apple. Blue and red circles (nodes) represent fungal and bacterial taxa, respectively. The core microbiome was calculated for each *Malus* group separately as amplicon sequence variants present in at least 70% of the samples. Node size corresponds to bacterial and fungal abundance, i.e. gene copy numbers measured by qPCR, as indicated in the legend on the lower left.

The apple core microbiome (defined as ASVs present in 70% of the replicates of each domestication group), comprised 18 fungal and 12 bacterial ASVs (Fig. 3c). These core taxa represented a high fraction of the total microbial community, measured via qPCR, with ASVs annotated to *Sphingomonas*, *Pseudomonas*, *Methylobacterium* and *Aureobasidium* being most abundant among all apple groups. Core fungi accounted for 75%, 62%, and 63% of total ITS copy numbers detected in wild, progenitor, and domesticated apple, respectively. Abundance of core bacteria was slightly less for the three domestication groups, representing 48%, 45%, and 49% of total 16S rRNA gene copy numbers, respectively. In addition to those shared ASVs, six bacterial ASVs were unique to the wild species core microbiome, three bacterial and one fungal ASVs were unique to progenitor species, and three bacterial and 18 fungal ASVs were only detected in the domesticated apple core microbiome. The fraction of the unique core taxa in wild species was < 0.01% for fungi and 4.2% for bacteria, in progenitor species it was 0.2% for fungi and 1.2% for bacteria, and in domesticated apple it was 21% for fungi and 2.6% for bacteria.

Within *M. domestica*, heirloom and modern cultivars did not differ significantly in their species richness (fungi: *P* = 0.999 and bacteria: *P* = 0.681) nor Shannon diversity (fungi: *P* = 0.832 and bacteria: *P* = 0.474) (Fig. 2). This was also true regarding community composition of their core microbiomes (fungi: *R* ^2^ = 0.047, *P* = 0.499 and bacteria: *R* ^2^ = 0.022, *P* = 0.869). Despite the lack of statistical differences, a hierarchical analysis revealed a clear distinction between the modern and heirloom cultivars, with two exceptions (Splendour and Miron Sacharanij), for both the fungal and bacterial core communities (Fig. 3a,b).

### The effect of phylogeny on the community composition of *Malus* species

**Fig. 4:**
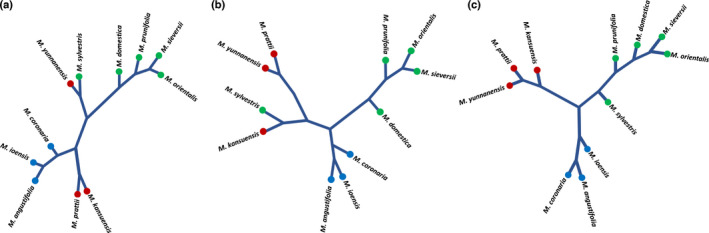
Results of hierarchical clustering based on Bray–Curtis dissimilarity distances of the fungal (a) and bacterial (b) community composition using clustering method ‘average’. (c) Shows the phylogenetic tree based on *Malus* ITS gene. *Malus* phylogenetic distance was inferred by using the neighbor‐joining tree estimation in R package phangorn. The leaf color indicates *Malus* groups: green = *Malus* × *domestica* and its wild progenitors (*M. sieversii*, *M. orientalis*, *M. prunifolia*, and *M. sylvestris*), blue = North American species (*M. angustifolia*, *M. coronaria*, and *M. ioensis*), and red = Asian species (*M. kansuensis*, *M. yunnanensis*, and *M. prattii*). The phylogenic plots were visualized using the *fviz_dend* function in the R package factoextra v.1.0.7.

Hierarchical clustering, based on Bray–Curtis dissimilarity metrics of the fungal and bacterial community composition, revealed that *M*. *domestica* and its progenitor species (*M. sieversii*, *M. prunifolia*, and *M. orientalis*) clustered separately from wild *Malus* species. The sole exception to this clustering was *M. sylvestris* which clustered with *M*. *yunnanensis* and *M. kansuensis* in the fungal and bacterial trees, respectively (Fig. 4a,b). This pattern of clustering largely corresponded with the phylogeny of *Malus* species (Fig. 4c). The correlation between the evolutionary distance of *Malus* species and their associated microbial communities was found to be significant for both fungal (*R* ^2^ = 0.471, *P* = 0.024) and bacterial (*R* ^2^ = 0.465, *P* = 0.035) communities, based on Procrustes analysis. The correlation between the phylogenetic distance and the core microbiome of each *Malus* species, however, was not significant for either fungi (*R* ^2^ = 0.5624, *P* = 0.119) or bacteria (*R* ^2^ = 0.297, *P* = 0.158).

### Estimating the origin of the *Malus* microbiome

**Fig. 5:**
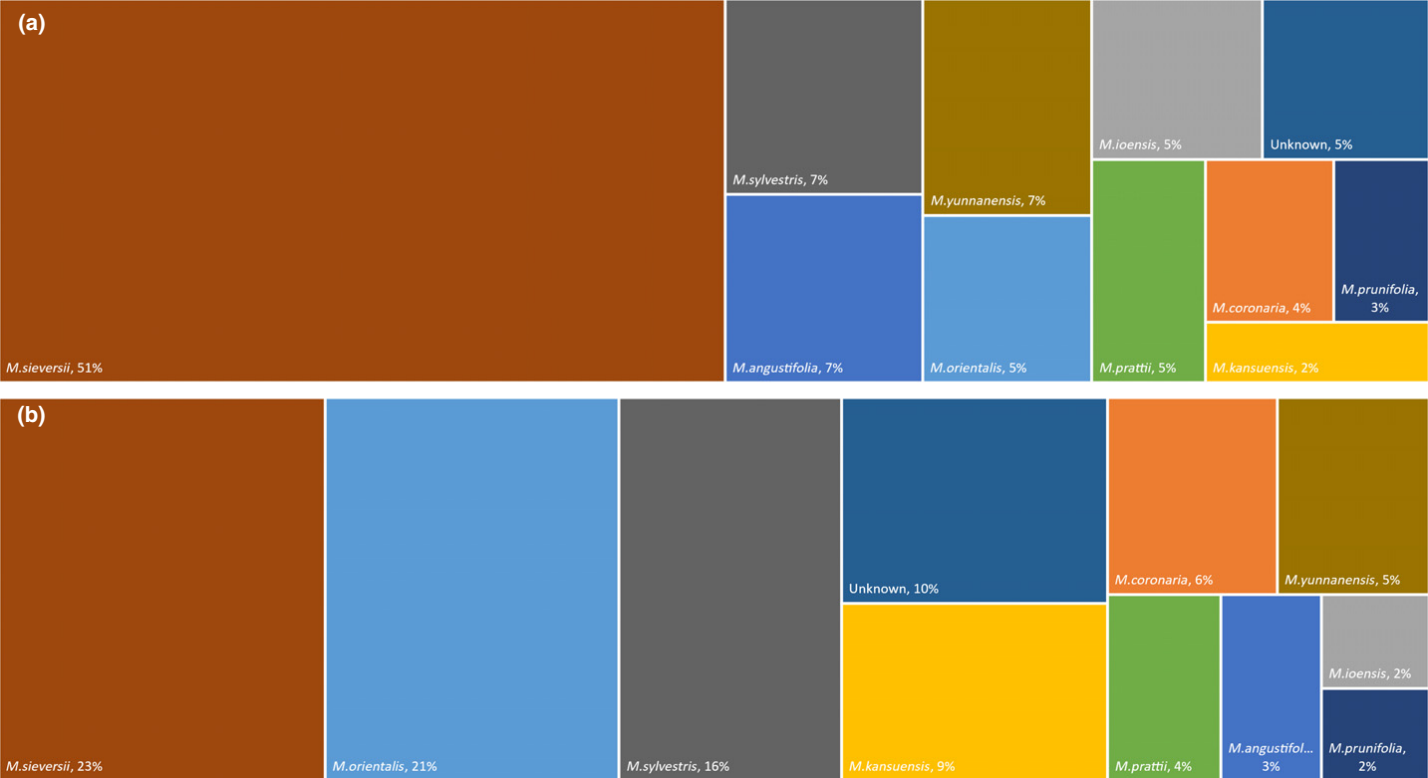
Treemap charts showing the estimated sources of the fungal (a) and bacterial (b) communities in *Malus domestica*. The estimates were calculated using Bayesian approach as implemented in SourceTracker2 by setting *M. domestica* as the sole sink and all the other *Malus* species (*M. sieversii*, *M. orientalis*, *M. prunifolia*, *M. sylvestris*, *M. kansuensis*, *M. yunnanensis*, *M. angustifolia*, *M. coronaria*, *M. ioensis*, and *M. prattii*) as potential sources. An unknown source was added automatically by the algorithm to allocate taxa in *M. domestica* with low probability to have originated from any of the assigned sources. The fungal and bacterial communities were rarefied to 1500 reads per sample in both the sink and sources.

The community‐wide Bayesian model estimated that the majority of the fungal community of *M. domestica* originated from its progenitor species *M. sieversii* (51%), with an equal contribution from *M. sylvestris* (7%), *M. yunnanensis* (7%), and wild *M. angustifolia* (7%), followed by *M. prattii* (5%), *M. ioensis* (5%), and *M. orientalis* (5%) (Fig. 5a). The three progenitor species *M. sieversii* (23%), *M. orientalis* (21%), and *M. sylvestris* (16%) were the main source of the *M*. *domestica* bacterial community (Fig. 5b). A detailed description of ASVs estimated to have contributed to *M. domestica* from other *Malus* species is presented in Fig. S3.

## Discussion

We examined the effects of domestication and species identity on the endophytic microbiome of *Malus*, including wild apple species, apple wild progenitors, and heirloom and modern cultivars of domesticated apple. We demonstrated (1) that the fungal community associated with domesticated apples and their wild progenitors had a higher species richness, diversity, abundance and distinct fungal and bacterial community compared to wild species, (2) strong evidence of phylosymbiosis for both fungal and bacterial communities, and (3) the microbiome of the domesticated apple to be an admixture of its wild progenitors with clear evidence of introgression for the bacterial community.

### Impact of domestication on microbial diversity and composition

We observed significantly higher fungal richness, diversity, and abundance in domesticated apple compared to its wild ancestors. While this contrasts with the common hypothesis that domestication reduces microbial diversity (Mutch & Young, 2004; Kiers *et al*., 2007; Leff *et al*., 2017; Pérez‐Jaramillo *et al*., 2018; Porter & Sachs, 2020; Favela *et al*., 2021), it is in agreement with some previous reports (Cardinale *et al*., 2015; Abdullaeva *et al*., 2020). The increase in fungal diversity indicates that domestication resulted in a greater rate of species gain than loss. However, when considering that beta diversity was mainly explained by species turnover patterns, it is apparent that species gain was accompanied by considerable species turnover. The higher species diversity might be due to an increased niche size or amount of resources in domesticated apple. The increase in the resource availability is supported by the qPCR assays, which showed that domesticated *Malus*, both heirloom and modern cultivars, had a significantly higher quantity of microbial cells than their wild relatives. It might be that plant domestication and breeding for desirable traits have indirectly facilitated an increase in microbial population size. This hypothesis fits the evidence that a focus during domestication and breeding on increased yield, fruit size, water and sugar content results in lower levels of defense chemicals and stress resistance (Cornille *et al*., 2014; Whitehead & Poveda, 2019; Porter & Sachs, 2020). Indeed, wild apples have been shown to contain higher total phenolic concentrations and a higher diversity of metabolites than domesticated apples and are known to be more resistant to major apple diseases (Ballester *et al*., 2017; Sun *et al*., 2017; Whitehead & Poveda, 2019; Singh *et al*., 2021).

The bacterial core microbiome appeared to be relatively stable along the chronosequence of *Malus* germplasm, i.e. from wild to wild progenitor, and from wild progenitor to domesticated apples. However, the number of fungal core species was significantly higher in *M*. *domestica* compared to wild species. Collectively, the core microbiome represented a large fraction of the microbial community of wild, progenitor, and domesticated apple, accounting for two‐thirds and almost half of the abundance of the fungal and bacterial communities, respectively. The existence of such core microbiome that spans the *Malus* phylogeny suggests an evolutionary conservation of the core microbiome and is in agreement with an ecological role across evolutionary boundaries (Yeoh *et al*., 2017). The mechanism(s) by which such a community is maintained across the *Malus* phylogeny is difficult to pinpoint without further studies. Nevertheless, we speculate that continuous transmission of the core microorganisms across generations through vertical transmission, along with positive selection on retention of the core microbiome, represents a plausible explanation (Gundel *et al*., 2011; Hodgson *et al*., 2014; Shade *et al*., 2017; Bergna *et al*., 2018; Shahzad *et al*., 2018; Kim *et al*., 2020; Abdelfattah *et al*., 2021b). Our hypothesis is supported by recent findings including: (1) similar core species were reported in a global survey of the apple fruit microbiome, and (2) the role of microbial inheritance (vertical transmission) is increasingly being recognized to play an essential role in the continuity of the plant microbiome (Saikkonen *et al*., 2002; Gundel *et al*., 2011; Hodgson *et al*., 2014; Shade *et al*., 2017; Bergna *et al*., 2018; Shahzad *et al*., 2018; Kim *et al*., 2020; Abdelfattah *et al*., 2021a, 2021b,2021a, 2021b).

### Evidence of phylosymbiosis

We found a significant correlation between the evolutionary distance of *Malus* species and their associated fungal and bacterial endophytic communities, which explained 47% of the observed differences. Based solely on the microbial community composition, we were able to distinguish between wild species from North America and Asia. However, the most intriguing result was the clustering of domesticated cultivars with their wild progenitors. These results demonstrate that phylosymbiosis exists in the genus *Malus*. Previous studies have reported phylosymbiosis between root‐associated bacterial communities and diverse groups of plants including lycopods, ferns, gymnosperms, and angiosperms (Bouffaud *et al*., 2014; Schlaeppi *et al*., 2014; Vincent *et al*., 2016), but, as far as we know, no such relationship has been reported for the fungal and bacterial endophytic community of *Malus*. These findings match the expectation that the genetic makeup of plants, driven by evolution and domestication, shapes the structure of the plant microbiome (Leff *et al*., 2017; Kim *et al*., 2020; Spor *et al*., 2020; Deng *et al*., 2021; Wagner, 2021). Conversely, the correlation between *Malus* phylogeny and the microbial community composition was not significant when considering the core microbiome. This is in agreement with the notion that rare taxa are important for structuring communities and for distinguishing between closely related plant species (Li *et al*., 2018; Ramirez *et al*., 2018; Berg *et al*., 2020). Rare taxa are also hypothesized to offer a pool of genetic resources that may be activated under the appropriate conditions (Jousset *et al*., 2017).

### The origin of the *M. domestica* microbiome

The results of the Bayesian approach showed that *M. sieversii*, the main ancestor of *M. domestica*, accounted for 51% and 23% of the fungal and bacterial communities of domesticated apple, respectively. These results are in strong agreement with the genetic origin of domesticated apple. Although the microbiome of *M*. *sylvestris* clustered separately from the other wild progenitors, the Bayesian model showed it had contributed 16% of the bacterial community of *M. domestica*. This could be explained by the fact that *Sphingomonas* and *Methylobacterium‐Methylorubrum*, two highly abundant ASVs in both *M. sylvestris* and *M. domestica* were identified by the algorithm to originate from *M. sylvestris*. Such findings indicate that introgressive hybridization that occurred during domestication between apple progenitors comprised both genetic and microbial features, including some of the most important genera in domesticated apple. Although genetic hybridization is known as the incorporation of alleles from one species into the gene pool of another, the mechanisms by which microbial hybridization occurs has not, to the best of our knowledge, been studied. However, early studies on breeding show that similar mechanisms could apply for the transmission and hybridization of the microbiome (Adam *et al*., 2018; Kusstatscher *et al*., 2021a; Sahu & Mishra, 2021).

### Evidence of co‐evolution

Whether plants and their microbiomes are co‐evolving or evolving together is a question that is still under debate (Theis *et al*., 2016; Limborg & Heeb, 2018). This is mainly because co‐evolution, in *sensu stricto*, is expected to result in reciprocal changes in the involved parties as, for example, in the case of pea‐aphid and its endosymbiotic bacteria *Buchnera* sp., whereby amino acid synthesis occurs through cooperation between host and symbiont (Brundrett, 2002; Russell *et al*., 2013). Another example, is the evolution of complementary traits between plants and mycorrhizal fungi, where fungi depend on the host plant carbon for energy consumption and hosts providing a more hospitable environment for fungi (Brundrett, 2002; Hoeksema, 2010). However, there is a growing body of literature that considers the microbiome as a superorganism and single unit of selection, in particular within the context of the holobiont framework (Theis *et al*., 2016; Ravanbakhsh *et al*., 2021; Tan *et al*., 2021). When taking this perspective, we think that the patterns of increased diversity with domestication, phylosymbiosis, and a strongly conserved core microbiome across the *Malus* phylogeny, together with evidence for concurrent plant and microbiome admixture and introgression, provide support for co‐evolution between *Malus* species and their microbiome during domestication. Yet, we caution that these patterns could also be explained by other, nonmutually exclusive mechanisms. For instance, phylosymbiosis can readily emerge from a simple ecological filtering process, where a host trait that varies with host phylogeny could act as a filter for preadapted microbes (Theis *et al*., 2016; Mazel *et al*., 2018; Beilsmith *et al*., 2019; Wagner, 2021). In which case, the metacommunity is also expected to play an important role since it represents the species pool of which plants and microorgamisns can establish their associations. To determine the underlying (co‐)evolutionary processes shaping the current patterns, we suggest that future studies use shotgun metagenomics and/or genome‐wide association studies to identify changes in the genetic composition of microbial species during domestication, combined with experiments that identify the functional role of any identified genetic variation.

### Conclusion

Several recent initiatives have called for intentional manipulation of the plant microbiome to enhance crop performance and sustainability, especially in the framework of sustainable agroecosystems (Berg, 2009; D’Hondt *et al*., 2021; Favela *et al*., 2021; French *et al*., 2021). The pattern of phylosymbiosis, and the congruent pattern of plant and microbial admixture, indicates that changes in the plant microbiome can be predicted, and further supports the idea that microbiome‐based breeding strategies are feasible (Adam *et al*., 2018; Chen *et al*., 2020; Favela *et al*., 2021; Kusstatscher *et al*., 2021b). Overall, the increase in diversity, phylogenetic correlation, and the estimated origin of the apple microbiome indicate strongly towards microbial introgression events that occurred during *Malus* domestication and supports co‐evolution between plants and their microbiomes.

## Author contributions

AA, JN, SD and MW conceptualized and designed the experiment. JL performed sample preparation and DNA extractions. BW performed and interpreted qPCR assays. AA analyzed and interpreted the amplicon data. AA wrote the first draft, and AJMT made a major contribution to the final version. GB, JN, SD, and MW contributed to the interpretation of the results and writing of the manuscript.

## Supporting information


**Fig. S1** Stacked bar charts showing the fungal (a) and bacterial (b) community composition of the most abundant taxa with a relative abundance > 0.1% across all the investigated *Malus* species.
**Fig. S2** Microbial gene copy numbers in apple shoots determined by qPCR and the estimated beta diversity partitioning.
**Fig. S3** Bar chart showing the estimated sources of the fungal (a) and bacterial (b) taxa in *Malus domestica* (modern apple).

**Table S1** Metadata table with the information regarding *Malus* accessions used in the present study.

**Table S2** List of ITS sequences used to calculate distances among *Malus* species and the phylogenetic trees.
**Table S3** Analysis of variance on the effect of domestication and *Malus* phylogeny (species) on fungal and bacterial diversity based on Shannon index.
**Table S4** Results of statistical pairwise‐comparisons of fungal and bacterial diversity based on Shannon index and community composition based on Bray–Curtis dissimilarity index using adonis (˜PERMANOVA).
Please note: Wiley Blackwell are not responsible for the content or functionality of any Supporting Information supplied by the authors. Any queries (other than missing material) should be directed to the *New Phytologist* Central Office.

## Data Availability

The datasets generated and/or analyzed during the current study are available in the ‘SRA NCBI’ repository, and can be accessed from the following link https://www.ncbi.nlm.nih.gov/bioproject/PRJNA702287. The code used in this study is available on zenodo and can be accessed from the following link https://doi.org/10.5281/zenodo.5578000.
