# Supplementary material for: Evidence for host–microbiome co‐evolution in apple
Source: New Phytol. 2021 Nov 25;234(6):2088–100. doi: 10.1111/nph.17820 (PMC9299473; doi:10.1111/nph.17820)

New Phytologist Supporting Information, Evidence for host-microbiome co-evolution in apple. Ahmed Abdelfattah, Ayco J.M. Tack, Birgit Wasserman, Jia Liu, Gabriele Berg, John Norelli, Samir Droby, Michael Wisniewski. 18 October 2021.

Figure S1 Stacked bar charts showing the fungal (a) and bacterial (b) community composition of the most abundant taxa with a relative abundance >0.1% across all the investigated *Malus* species *M. domestica, M. sieversii, M. orientalis, M. prunifolia, and M. sylvestris M. kansuensis, M. yunnanensis, M. angustifolia, M. coronaria, M. ioensis,* and *M. prattii.*

a

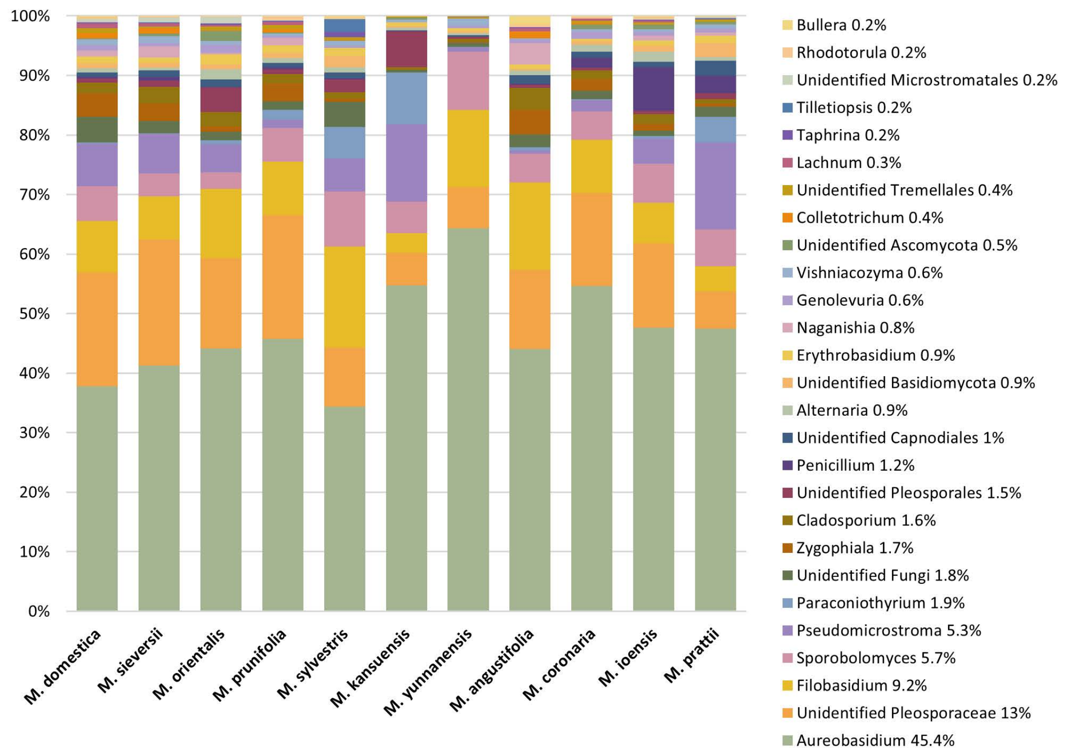

b

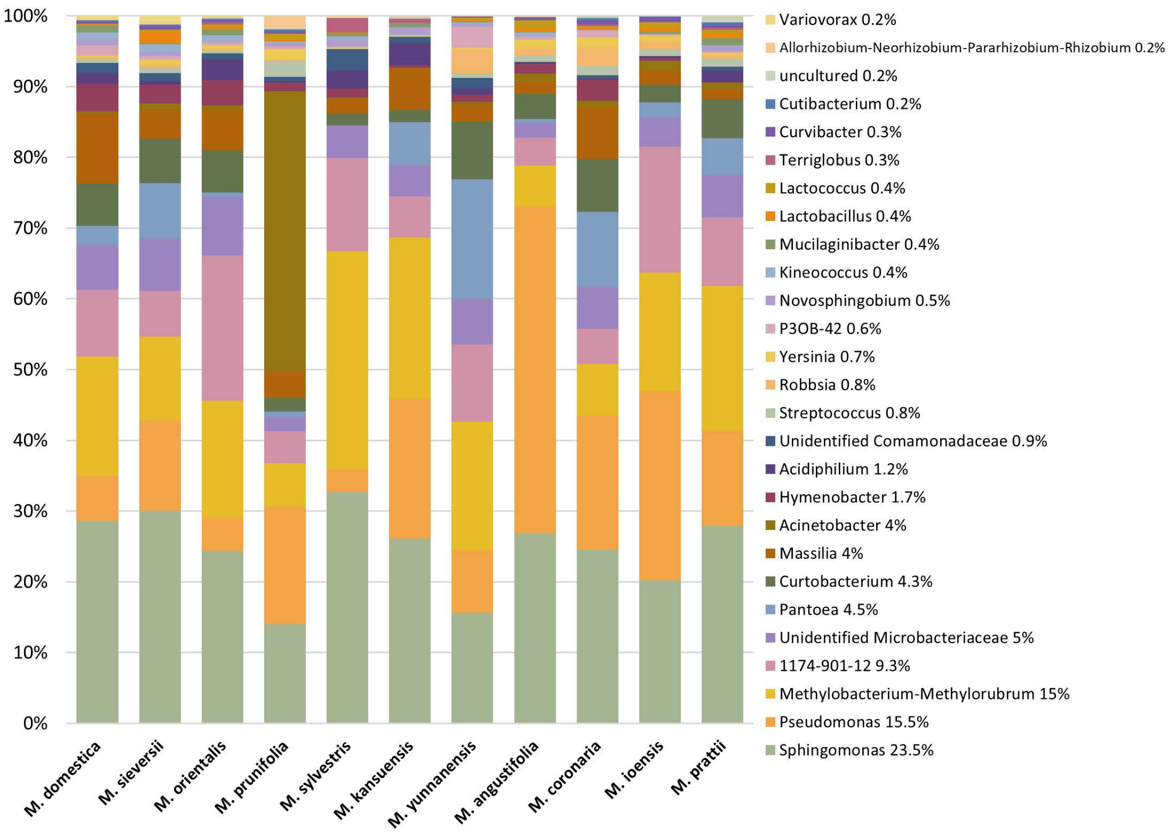

Figure S2 Microbial gene copy numbers in apple shoots determined by qPCR. Values are given by primers targeting fungal ITS region (a) and bacterial 16S rRNA genes (b). Gene copy numbers are calculated per shoot used to extract DNA for microbiome analysis and values for individual sample replicates were summarized for the three apple domestication groups (domesticated, progenitors, wild). Box plots show the median (horizontal line), the lower and upper bounds of each box plot denote the first and third quartiles, and whiskers above and below the box plot show 1.5 times the interquartile range. The points located outside of the whiskers of the box plot represent the outliers. Asterisks indicate significant difference in microbial gene copy number abundance between groups, calculated by non-parametric Kruskal-Wallis test and the p-values were corrected using Bonferroni multiple test correction. Empty circles indicate outliers. Estimated beta diversity partitioning the three domestication groups between species turnover (JTU) and nestedness (JNE) for the fungal (c) and bacterial community (d).

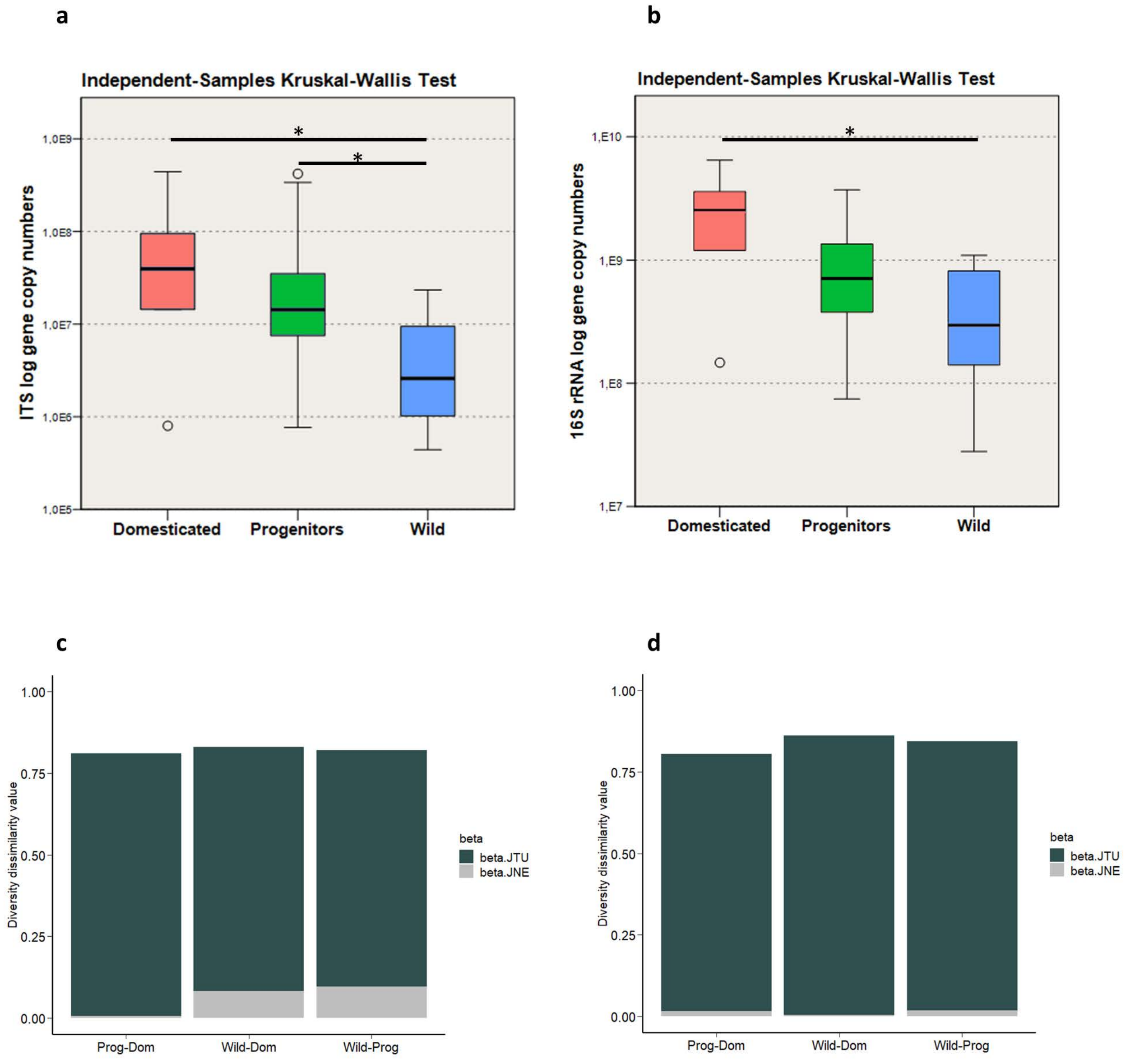

Figure S3 Bar chart showing the estimated sources of the fungal (a) and bacterial (b) taxa in *Malus domestica* (modern apple). The estimates were calculated using Bayesian approach as implemented in Sourcetracker2 by setting *Malus domestica* as the sole sink and all the other *Malus* species (*M. sieversii, M. orientalis, M. prunifolia, M. sylvestris, M. kansuensis, M. yunnanensis, M. angustifolia, M. coronaria, M. ioensis,* and *M. prattii*) as potential sources, as well as the unknown source added automatically by the algorithm. The fungal and bacterial communities were rarefied to 1500 reads per sample in both the sink and sources.

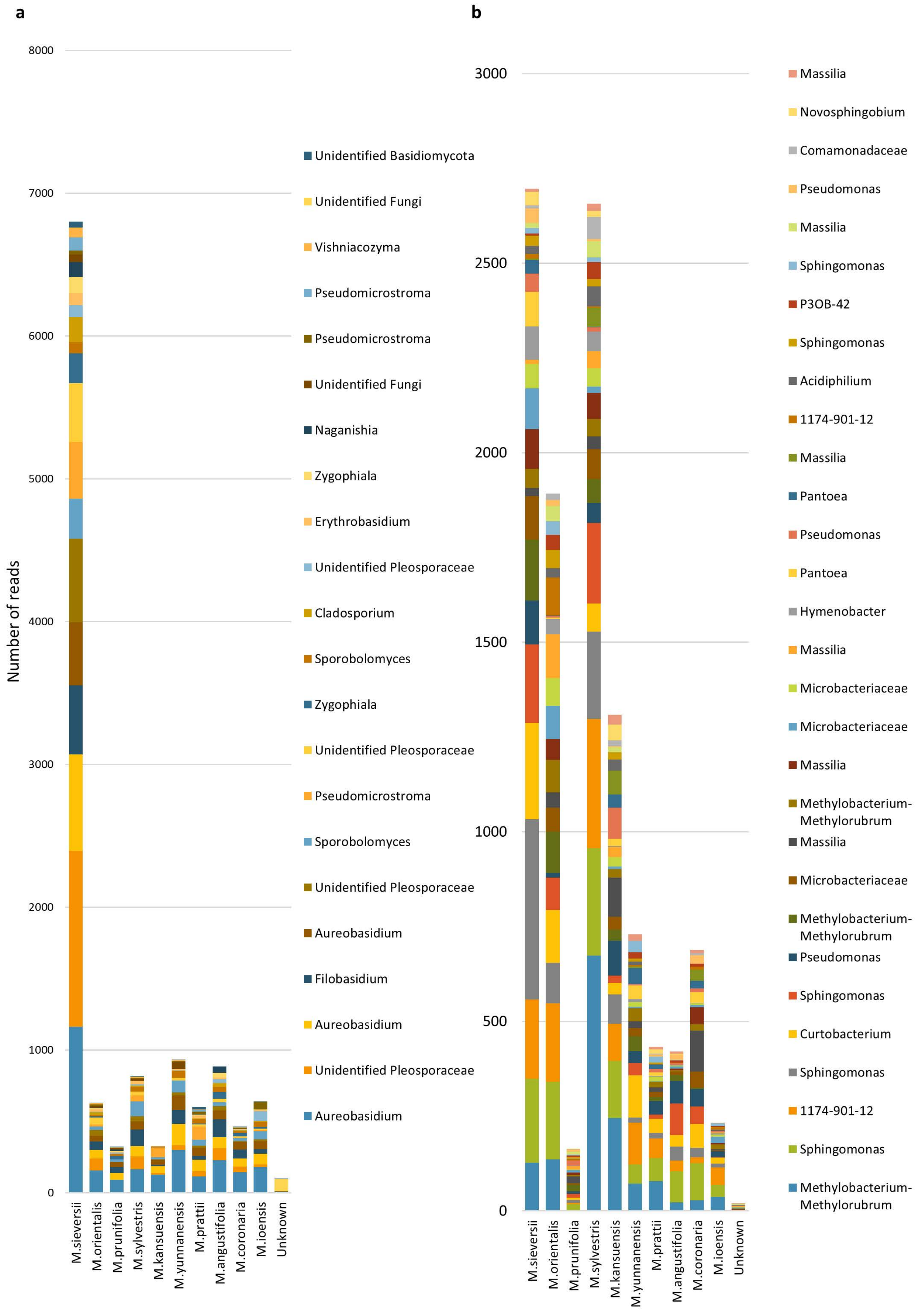

Supplement: Supplementary file 1 — Fig. S1 Stacked bar charts showing the fungal (a) and bacterial (b) community composition of the most abundant taxa with a relative abundance > 0.1% across all the investigated Malus species. Fig. S2 Microbial gene copy numbers in apple shoots determined by qPCR and the estimated beta diversity partitioning. Fig. S3 Bar chart showing the estimated sources of the fungal (a) and bacterial (b) taxa in Malus domestica (modern apple). [file NPH-234-2088-s003.pdf]
