# Supplementary material for: Evidence for host–microbiome co‐evolution in apple
Source: New Phytol. 2021 Nov 25;234(6):2088–100. doi: 10.1111/nph.17820 (PMC9299473; doi:10.1111/nph.17820)
Supplement: Supplementary file 3 — Table S2 List of ITS sequences used to calculate distances among Malus species and the phylogenetic trees. Table S3 Analysis of variance on the effect of domestication and Malus phylogeny (species) on fungal and bacterial diversity based on Shannon index. Table S4 Results of statistical pairwise‐comparisons of fungal and bacterial diversity based on Shannon index and community composition based on Bray–Curtis dissimilarity index using adonis (˜PERMANOVA). Please note: Wiley Blackwell are not responsible for the content or functionality of any Supporting Information supplied by the authors. Any queries (other than missing material) should be directed to the New Phytologist Central Office. [file NPH-234-2088-s002.pdf]

New Phytologist Supporting Information, Evidence for host-microbiome co-evolution in apple. Ahmed Abdelfattah, Ayco J.M. Tack, Birgit Wasserman, Jia Liu, Gabriele Berg, John Norelli, Samir Droby, Michael Wisniewski. 18 October 2021.

Table S2. The supplementary document includes the accession and sequences of Malus species, the calculated distances, and the phylogenetic trees.

>EF113171.1 Malus kansuensis
TCGAACCTGCACAGCAGAACGACCCGAGAACCCGTTTCAACGTCGGGGGTCGGCGGGCCTCCGGGCCCGG
CGTCCCCTTTGTCCCGGGAGCTCGCTCCCGGGCGTACAAACGAACACCGGCGCGTATTGCGCCAAGGAAC
CTGAACGAAAGAGAGCGCTCCCGCCGCCCGGAAACGGTGTGCGAGCGGGTGCGTCGTCTTCTTCGATATG
TCAAAACGACTCTCGGCAACGGATATCTCGGCTCTCGCATCGATGAAGAACGTAGCGAAATGCGATACTT
GGTGTGAATTGCAGAATCCCGTGAACCATCGAGTCTTTGAACGCAAGTTGCGCCCGAAGCCATCAGGCCG
AGGGCACGCCTGCCTGGGCGTCACACGCCGTTGCCCCCCCCGCACCTCCTTAGGGAGCGTCGGAGGGGCG
GAAGATGGCCTCCCGTGCGTCACCCCGCGCGGTTGGCACAAAAGCCGGGTCCTCGGCGACGAACGCCACG
ACAATCGGTGGTTGTCGAACCTCGGTTGCCCGTTGTGCGCTTTCGTCGCGGTCCGAGCGGCTCGCGACGC
TCACTGCTCCGCTTCGACGGAGCTTTCAACG

>JQ392466.1 Pyrus communis
TTGTCGAACCTGCACAGCAGAATGACTCGAGAACCTATTCCAATGTCGAGGGCCGGCGGGCCTTCAAGCT
TGGCGTCCCCTTATCCCGGAAGCCCGCTCCTGGGCGTACAAACGAACACCGGTGCGTCTTACGCCAAGGA
ACCTAAACGAAAGAGCGCGCTCCAGCCACCTCGGAAACAGTGCACGCGCGAGTGCGTAGTCGTCTTCTAT
AAGTCAAAATGACTCTCAGTAACGGATATCTCGGCTCTCGTATCGATCAAGAACGTAGCGAAATGCAATA
CTTGGTGTGAATTGCAGAATCCCGTGAACCATCGAGTCTTTGAACACAAGTTGCGAGGTCATGCTTGCCT
AGGAGTCACACGTCGTTGCCCCCCTGACACCTTCCTCGGGAGCGTCGGGGTGCAGACGATATCCTCCTGT
GCATCGACCTGCGCGATTGGCATAAATGTCGAGTCCTCGACGACGAATGCCAAGACAATCGATGGTTACC
AAACCTCGGTTGCCTGTTGTGCGCTTTCGTCCCGCTCCGAGTGGCTCGCGACGCTCCCCGCTTTACTTCG
GCCAAGCTTTCAACA

>MH270485.1 Malus yunnanensis
TCGAACCTGCACAGCAGAACGACCCGAGAACCCGTTTCAACGTCGGGGGAACGGCGGGCCTCCGGGCCCG
GCGTCCCCTTTGTCCCGGGACCCCGCTCCCGGGCGTACAAACGAACACCGGCGCGTGTTGCGCCAAGGAA
CCTGAACGAAAGAGAGCGCTCCCGCCGCCCCGTAAACGGTGCGCGAGCGGGTGCGTCGTCTTCTTCGATA
TGTCAAAACGACTCTCGGCAACGGATATCTCGGCTCTCGCATCGATGAAGAACGTAGCGAAATGCGATAC
TTGGTGTGAATTGCAGAATCCCGTGAACCATCGAGTCTTTGAACGCAAGTTGCGCCCGAAGCCATCAGGC
CGAGGGCACGCCTGCCTGGGCGTCACACGCCGTTGCCCCCCCCGCACCTCCCTCGGGAGCGTCGGAGGGG
CAGAAGATGGCCTCCCGTGCGTCACCCCGCGCGGTTGGCACAAAAGTCGGGTCCTCGGCGACGAACGCCA
CGACAATCGGTGGTTGTCAAACCTCGGTTGCCCGTTGTGCGCTTTCGTCGCGCTTCGAGCGGCTCGCGAT
GCTCACTGCTCCGCTTCGGCGGAGCTTTCAACGCGA

>JQ392445.1 Malus prattii
TTGTCGAACCTGCACAGCAGAACGACCCGAGAACCCGTTTCAACGTCGGGGGAACGGCGGGCCTCCGGGC
CCGGCGTCCCCTTTGTCCCGGGAGCCCGCTCCCGGGCGTACAAACGAACACCGGCGCGTGTTGCGCCAAG
GAACCTGAACGAAAGAGAGCGCTCCCGCCGCCCCGTAAACGGTGCGCGGGCGGGTGCGTCGTCTTCTTCG

ATATGTCAAAACGACTCTCGGCAACGGATATCTCGGCTCTCGCATCGATGAAGAACGTAGCGAAATGCGA
TACTTGGTGTGAATTGCAGAATCCCGTGAACCATCGAGTCTTTGAACGCAAGTTGCGCCCGAAGCCATCA
GGCCGAGGGCACGCCTGCCTGGGCGTCACACGCCGTTGCCCCCCCCGCACCTCCCTCGGGAGCGTCGGAG
GGGCRGAAGATGGCCTCCCGTGCGTCACCCCGCGCGGTTGGCACAAAAGTCGGGTCCTCGGCGACGAACG
CCACGACAATCGGTGGTTGTCAAACCTCGGTTGCCCGTTGTGCGCTTTCGTCGCGCTTCGAGCGGCTCGC
GATGCTCCCTGCTCCGCTTCGGCGGAGCTTTCAACG

>JQ392448.1 Malus ioensis
TTGTCGAACCTGCGCAGCAGAACGACCCGAGAACCAGTTTCAAAGTCGGGGGTCGGCGGGCCTCCGGGCT
CGGCGTCCCCTTCGTCCCGGGAGCCCGCTCCCGGGCGTACAAACGAACACCGGCGCGAACTGCGCCAAGG
AACCTGAACGAAAGAGCGCGCTCCCGCCGCCCCGGAAACGGTGCGCGCGCGGGTGCGTCGTCGTCTTCCA
TACAAGTCAAAACGACTCTCGGCAACGGATATCTCGGCTCTCGCATCGATGAAGAACGTAGCGAAATGCG
ATACTTGGTGTGAATTGCAGAATCCCGTGAACCATCGAGTCTTTGAACGCAAGTTGCGCCCGAAGCCATC
AGGCCGAGGGCACGCCTGCCTGGGCGTCACACGCCGTTGCACCCCCGCACCTCCCTCGGGAGCGTCGGGG
GGCGGACGATGGCCTCCCGCGCGTCACCCCGCGCGGTTGGCACAAATGCCGGGTCCTCGGCGACGGACGC
CACGACAATCGGTGGTCGTCGAACCTCGGTTGCCCGTTGTGCGCTTTCGTCGCGCCCCGAGCGGCCCGCG
ACCCTCGCTGCTCCCGCTTCGGCGGAGCTTTCGACG

>AF186525.1 Malus coronaria
TCGAACCTGCGCAGCAGAACGACCCGAGAACCAGTTTCAAAGTCGGGGGTCGGCGGGCCTCCGGGCTCGG
CGTCCCCTTTGTCCCGGGAGCCCGCTCCCGGGCGTACAAACGAACACCGGCGCGAACTGCGCCAAGGAAC
CTGAACGAAAGAGCGCGCTCCCGCCGCCCCGGAAACGGTGCGCGCGCGGGTGCGTCGTCGTCTTCCATAC
AAGTCAAAACGACTCTCGGCAACGGATATCTCGGCTCTCGCATCGATGAAGAACGTAGCGAAATGCGATA
CTTGGTGTGAATTGCAGAATCCCGTGAACCATCGAGTCTTTGAACGCAAGTTGCGCCCGAAGCCATCAGG
CCGAGGGCACGCCTGCCTGGGCGTCACACGCCGTTGCCCCCCCGCACCTCCCTCGGGAGCGTCGGGGGGC
GGACGATGGCCTCCCGTGCGTCACCCCGCGCGGTTGGCACAAATGCCGGGTCCTCGGCGACGGACGCCAC
GACAATCGGTGGTCGTCGAACCTCGGTTGCCCGTTGTGCGCTTTCGTCGCGCCCCGAGCGGCCCGCGACC
CTCGCTGCTCCCGCTTCGGCGGAGCTTTCGACG

>AF186523.1 Malus angustifolia
TCGAACCTGCGCAGCAGAACGACCCGAGAACCAGTTTCAAAGTCGGGGGTCGGCGGGCCTCCGGGCTCGG
CGTCCCCTTCGTCCCGGGAGCCCGCTCCCGGGCGTACAAACGAACACCGGCGCGAACTGCGCCAAGGAAC
CTGAACGAAAGAGCGCGCTCCCGCCGCCCCGGAAACGGTGCGCGCGCGGGTGCGTCGTCGTCTTCSATAC
AAGTCAAAACGACTCTCGGCAACGGATATCTCGGCTCTCGCATCGATGAAGAACGTAGCGAAATGCGATA
CTTGGTGTGAATTGCAGAATCCCGTGAACCATCGAGTCTTTGAACGCAAGTTGCGCCCGAAGCCATCAGG
CCGAGGGCACGCCTGCCTGGGCGTCACACGCCGTTGCCCCCCCGCACCTCCCTCGGGAGCGTCGGGGGGC
GGACGATGGCCTCCCGTGCGTCACCCCGCGCGGTTGGCACAAATGCCGGGTCCTCGGCGACGGACGCCAC
GACAATCGGTGGTCGTCGAACCTCGGTTGCCCGTTGTGCGCTTTCGTCGCGCCCCGAGCGGCCCGCGACC
CTCGCTGCTCCCGCTTCGGCGGAGCTTTCGACG

>MK386850.1 Malus orientalis
TCGAACCTGCACGGCAGAACGACCCGAGAACCCGTTTCAACGTCGGGGGTCGGCGGGCCTCCTGGCCCGG
CGTCCCCTTCGTCCCGGGAGCCCGCTCCCGGGCGTACAAACTTACACCGGCGCGTGTTGCGCCAAGGAAT
CTGAACGAAAGAGCGCGCTCCCGCCGCCCCGGAATCGGTGCGCGCGCGGGTGCGTCGTCGTCTTCGATAA

GTCAAAACGACTCTCGGCAACGGATATCTCGGCTCTCGCATCGATGAAGAACGTAGCGAAATGCGATACT
TGGTGTGAATTGCAGAATCCCGTGAACCATCGAGTCTTTGAACGCAAGTTGCGCCCGAAGCCATCAGGCC
GAGGGCACGCCTGCCTGGGCGTCACACGCCGTTGCCCCCCCGCGCATCCCTCGGGAGCGTCGGGGGGCGG
ACGATGGCCTCCCGTGCTTCACCCCGCGCGGTTGGCCCAAATGCCGGGTCCTCGGCGACGAACGCCACGA
CAATCGGTGGTCGTCGAACCTCGGTTGCCAGTTGTGCGCTTTCGTCGCGCTCCGAGCGGCCCGCGACGCC
CGCTGCTCTCGCTTCGGCGGAGCTTTCGACG

>AF186492.1 Malus sieversii
TCGAACCTGCACGGCAGAACGACCCGAGAACCCGTTTCAACTTCGGGGGTCGGCGGGCCTCCTGGCCCGG
CGTCCCCTTCGTCCCGGGAGCCCGCTCCCGGGCGTACAAACTTACACCGGCGCGTGTTGCGCCAAGGAAT
CTGAACGAAAGAGCGCGCTCCCGCCGCCCCGGAATCGGTGCGCGCGCGGGTGCGTCGTCGTCTTCGATAA
GTCAAAACGACTCTCGGCAACGGATATCTCGGCTCTCGCATCGATGAAGAACGTAGCGAAATGCGATACT
TGGTGTGAATTGCAGAATCCCGTGAACCATCGAGTCTTTGAACGCAAGTTGCGCCCGAAGCCATCAGGCC
GAGGGCACGCCTGCCTGGGCGTCACACGCCGTTGCCCCCCCGCGCATCCCTCGGGAGCGTCGGGGGGCGG
ACGATGGCCTCCCGTGCTTCACCCCGCGCGGTTGGCCCAAATGCCGGGTCCTCGGCGACGAACGCCACGA
CAATCGGTGGTCGTCGAACCTCGGTTGCCAGTTGTGCGCTTTCGTCGCGCTCCGAGCGGCCCGCGACGCC
CGCTGCTCTCGCTTCGGCGGAGCTTTCAACG

>AF186478.1 Malus domestica
TCGAACCTGCACGGCAGAACGACCCGAGAACCCGTTTCAACTTCGGGGGTCGGCGGGCCTCCTGGCCCGG
CGTCCCCTTCGTCCCGGGAGCCCGCTCCCGGGCGTACAAACTTACACCGGCGCGTGTTGCGCCAAGGAAT
CTGAACGAAAGAGCGCGCTCCCGCCGCCCCGGAAWCGGTGCGCGCGCGGGTGCGTCGTCGTCTTCGATAA
GTCAAAACGACTCTCGGCAACGGATATCTCGGCTCTCGCATCGATGAAGAACGTAGCGAAATGCGATACT
TGGTGTGAATTGCAGAATCCCGTGAACCATCGAGTCTTTGAACGCAAGTTGCGCCCGAAGCCATCAGGCC
GAGGGCACGCCTGCCTGGGCGTCACACGCCGTTGCCCCCCCGCGCATCCCTCGGGAACGTCGGGGGGCGG
ACGATGGCCTCCCGTGCGTCACCCCGCGCGGTTGGCCCAAATGCCGGGTCCTCGGCGACGAACGCCACGA
CAATCGGTGGTCGTCGAACCTCGGTTGCCAGTTGTGCGCCTTCGTCGCGCTCCGAGCGGCCCGCGACGCC
CGCTGCTCTCGCTTCGGCGGAGCTTTCAACG

>JQ392462.1 Malus sylvestris
TTGTCGAACCTGCAAGGCAGAACGACCCGAGAACCCGTTTCAACGTTGGGGGTCGGCGGGCCTCCGGGCC
CGGCGTCCCCTTCGTCCCGGGAGCCCGCTCCCGGGCGTACAAACTTACACCGGCGCGTGTTGCGCCAAGG
AATATGAACGAAAGAGCGCGCTCCCGTCGCCCCGGAAACGGTGCGCGCGCGGGTGCGTCGTCGTCTTCGA
TAAAAGTCAAAACGACTCTCGGCAACGGATATCTCGGCTCTCGCATCGATGAANAACGTAGCGAAATGCG
ATACTTGGTGTGAATTGCAGAATCCCGTGAACCATCGAGTTTTTGAACGCAAGTTGCGCCCGAAGCCATC
AGGCCGAGGGCACGCCTGCCTGGGCGTCACACGCCGTTGCCCCCCCCGCGCATCCCTCGGGAGGGTCGGG
AGGGGGCGGACGATGNCCTCCCGTGCGTCACCCCGCGCGGTTGGAATAAATGCCGGGTCCTCGGCGACGA
ACGCCACGACAATTGGTGGTCGTCGAACCTCGGTTGCCAGTTGTGTGCTTTCGCCGCGCTCCGAGTGGCC
CGAGACGCTTGCTGCTCTTGCTTCGGCGTAGCTTTCAACG

>JQ392463.1 Malus prunifolia
TTGTCGAACCTGCACGGCAGAACGACCCGAGAACCCGTTTCAACGTCGGGGGTCGGCGGGCCTCCTGGCC
CGGCGTCCCCTTCGTCCCGGGAGCCCGCTCCCGGGCGTACAAACTTACACCGGCGCGTGTTGCGCCAAGG
AATCTGAACGAAAGAGCGCGCTCCCGCCGCCCCGGAAACGGTGCGCGCGCGGGTGCGTCGTCGTCTTCGA

TAAGTCAAAACGACTCTCGGCAACGGATATCTCGGCTCTCGCATCGATGAAGAACGTAGCGAAATGCGAT
ACTTGGTGTGAATTGCAGAATCCCGTGAACCATCGAGTCTTTGAACGCAAGTTGCGCCCGAAGCCATCAG
GCCGAGGGCACGCCTGCCTGGGCGTCACACGCCGTTGCCCCCCCGCGCATCCCTCGGGAGCGTCGGGGGG
CGGACGATGGCCTCCCGTGCGTCACCCCGCGCGGTTGGCACAAATGCCGGGTCCTCGGCGACGAACGCCA
CGACAATCGGTGGTCGTCGAACCTCGGTTGCCAGTTGTGCGCYTTCGTCGCGCTCCGAGCGGCCCGCGAC
GCTCGCTGCTCTCGCTTCGGCGGAGCTTTCAACG

[1] #EF113171.1_M. kansuensis [2] #JQ392462.1_M. sylvestris [3] #MH270485.1_M. yunnanensis [4] #JQ392445.1_M. prattii [5] #JQ392448.1_M. ioensis [6] #AF186525.1_M. coronaria [7] #AF186523.1_M. angustifolia [8]#MK386850.1_M. orientalis [9] #AF186492.1_M. sieversii [10]#AF186478.1_M. domestica [11] #JQ392463.1_M. prunifolia

[1	2	3	4	5	6	7	8	9	10	11]
[1]										
[2]	0.0732									
[3]	0.0258	0.075								
[4]	0.0259	0.0714	0.0051							
[5]	0.0529	0.0692	0.0584	0.0548						
[6]	0.0475	0.0673	0.0529	0.0493	0.0051					
[7]	0.0475	0.0637	0.0529	0.0494	0.0034	0.0017				
[8]	0.0549	0.0383	0.0567	0.0531	0.0456	0.0437	0.0402			
[9]	0.0549	0.0384	0.0567	0.0531	0.0492	0.0474	0.0438	0.0034		
[10]	0.0549	0.0384	0.0567	0.0531	0.0492	0.0474	0.0438	0.0085	0.0051	
[11]	0.0458	0.0295	0.0475	0.044	0.0402	0.0384	0.0349	0.0085	0.0085	0.0068

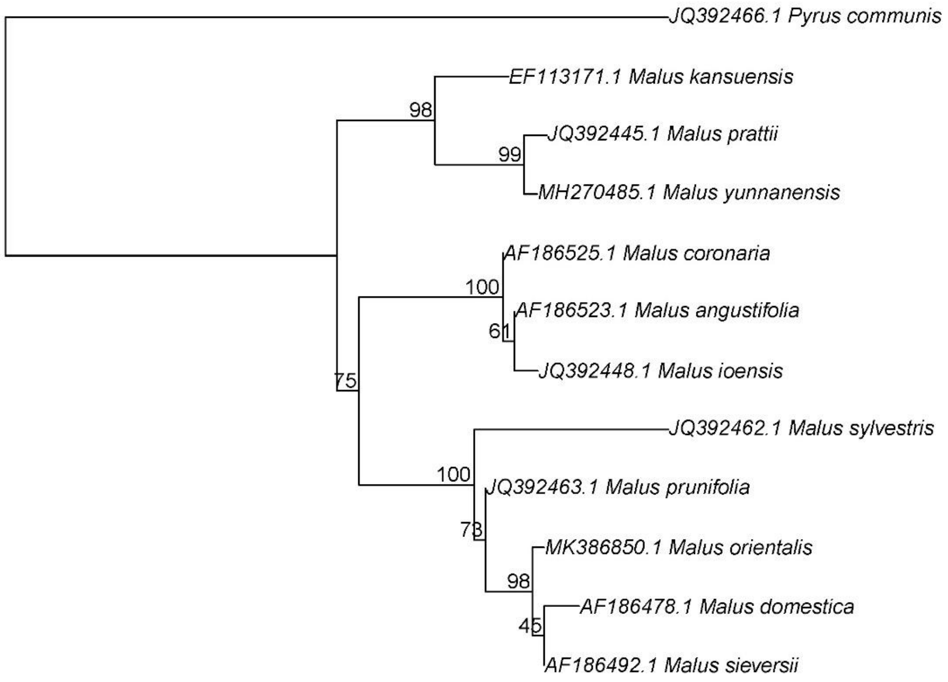

Table S3 Analysis of Variance on the effect of domestication and Malus phylogeny (species) on fungal and bacterial diversity based on Shannon index. In "Domestication" compares the three Domestication groups i.e. M. domestica, apple progenitor species (M. sieversii, M. orientalis, M. prunifolia, and M. sylvestris), and non-progenitor Malus species (M. kansuensis, M. yunnanensis, M. angustifolia, M. coronaria, M. ioensis, M. prattii). Degrees of freedom for domestication was 2 and for species 8.

	Fungi				Bacteria			
	Domestication		Species		Domestication		Species	
	F	*P*	*F*	*P*	*F*	*P*	*F*	*P*
Richness	9.143	0.0048	1.513	0.178	0.143	0.867	0.856	0.559
Evenness	1.470	0.2399	2.132	0.048	2.213	0.120	1.833	0.093
Diversity (Shannon)	5.004	0.0012	1.394	0.071	0.686	0.508	0.906	0.519

Table S4 Results of statistical pairwise-comparisons of fungal and bacterial diversity based on Shannon index and community composition based on Bray-Curtis dissimilarity index using adonis (~Permanova). P values were adjusted using FDR method for both tests. The test was done to compare the three Domestication groups i.e. M. domestica, apple progenitor species (M. sieversii, M. orientalis, M. prunifolia, and M. sylvestris), and wild Malus species (M. kansuensis, M. yunnanensis, M. angustifolia, M. coronaria, M. ioensis, M. prattii).

		Permanova		Shannon	Richness	Evenness
		R^2	*P*	*P*	*P*	*P*
Fungi	Wild vs Progenitors	0.033	0.038	0.003	0.004	0.272
	Wild vs Domesticated	0.053	0.006	0.006	0.001	0.566
	Progenitors vs Domesticated	0.033	0.038	0.983	0.757	0.911
Bacteria	Wild vs Progenitors	0.047	0.003	0.459	0.890	0.561
	Wild vs Domesticated	0.062	0.003	0.528	0.980	0.160
	Progenitors vs Domesticated	0.029	0.123	0.998	0.966	0.594
